# Function-based classification of hazardous biological sequences: Demonstration of a new paradigm for biohazard assessments

**DOI:** 10.3389/fbioe.2022.979497

**Published:** 2022-10-07

**Authors:** Bryan T. Gemler, Chiranjit Mukherjee, Carrie A. Howland, Danielle Huk, Zachary Shank, Lela Johnson Harbo, Omar P. Tabbaa, Craig M. Bartling

**Affiliations:** Battelle Memorial Institute, Columbus, OH, United States

**Keywords:** biohazard, sequence screening, virulence factor, biosecurity, biosafety

## Abstract

Bioengineering applies analytical and engineering principles to identify functional biological building blocks for biotechnology applications. While these building blocks are leveraged to improve the human condition, the lack of simplistic, machine-readable definition of biohazards at the function level is creating a gap for biosafety practices. More specifically, traditional safety practices focus on the biohazards of known pathogens at the organism-level and may not accurately consider novel biodesigns with engineered functionalities at the genetic component-level. This gap is motivating the need for a paradigm shift from organism-centric procedures to function-centric biohazard identification and classification practices. To address this challenge, we present a novel methodology for classifying biohazards at the individual sequence level, which we then compiled to distinguish the biohazardous property of pathogenicity at the whole genome level. Our methodology is rooted in compilation of hazardous functions, defined as a set of sequences and associated metadata that describe coarse-level functions associated with pathogens (e.g., adherence, immune subversion). We demonstrate that the resulting database can be used to develop hazardous “fingerprints” based on the functional metadata categories. We verified that these hazardous functions are found at higher levels in pathogens compared to non-pathogens, and hierarchical clustering of the fingerprints can distinguish between these two groups. The methodology presented here defines the hazardous functions associated with bioengineering functional building blocks at the sequence level, which provide a foundational framework for classifying biological hazards at the organism level, thus leading to the improvement and standardization of current biosecurity and biosafety practices.

## Introduction

**FIGURE 1:**
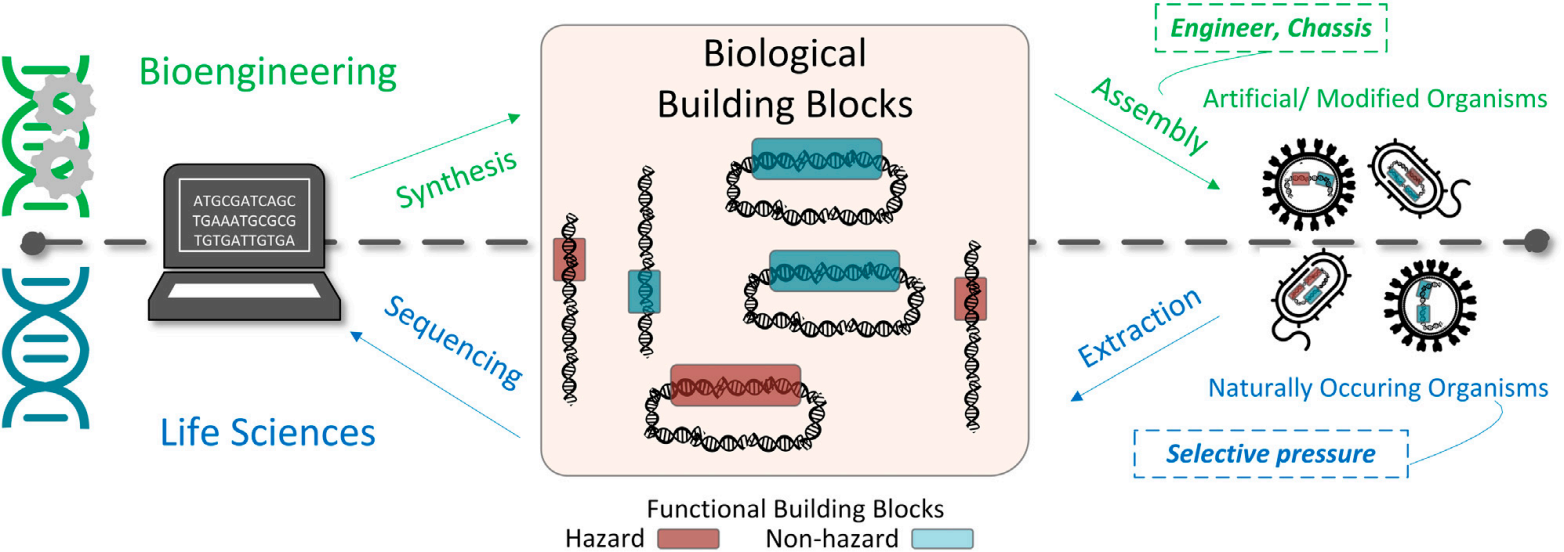
Understanding the hazard posed by bioengineering requires the characterization of hazardous building blocks. Bioengineering enables the creation of novel or modified organisms through oligonucleotide synthesis and assembly of building blocks within a biological vehicle. These biological functional building blocks are inspired from sequences extracted and sequenced from natural organisms. Such organisms can be composed of hazardous and non-hazardous functional elements (denoted by the red and blue blocks, respectively). The hazardous functions within organisms may have arisen from environmental selective pressures such as virus mutation to enable, for example, a jump from a vector to a human host, or a transfer of transposable elements among species.

The rapidly emerging discipline of bioengineering is enabling practitioners to analyze and assemble biological materials and microorganisms for industrial and research purposes through the creation of modified or novel organisms with specific functionalities (Slusarczyk et al., 2012). Bioengineering leverages sequences inspired from natural organisms that have been identified through studies in the life sciences (Figure 1). Exemplar chassis, such as *Escherichia coli* have been engineered with numerous functions, such as those to sense other bacteria, breakdown biofilms, and release toxic payloads (Hwang et al., 2017). While bioengineering is resulting in great benefit to mankind through medical advancements (e.g., precision medicine) and industrial use, the rapid progression and democratization of biotechnologies have presented new challenges for traditional biosafety and biosecurity practices.^1^ Current biosafety practices often focus on organisms at the species level, instead of the functional level, which hinders the ability to predict and accurately prepare for previously uncharacterized organisms, such as biodesigns (i.e., engineered organisms) with novel functionalities. For example, focused by a selected list of pathogens, appropriate laboratory safeguards can be put in place using Biosafety Levels promoted by the Centers for Disease Control and Prevention (CDC), which are based on the severity of the disease and infectivity of the organism being manipulated (U.S. Department of Health and Human Services, 2014). While useful in the current paradigm, these biosafety practices do no enable objective and clear guidelines for engineered organisms outside of prioritized lists of species.

Beyond laboratory safety, frameworks to bolster biosafety practices are in place in some countries for research approval (US Department of Health and Human Services, 2017) and DNA ordering (US Department of Health and Human Services, 2022). Current DNA screening practices used by the International Gene Synthesis Consortium (IGSG) follow a uniform screening protocol against a Restricted Pathogen Database (RPD) “derived from international pathogen and toxin sequence databases” (International Gene Synthesis Consortium, 2017). While practical for regulated pathogens, screening sequences against the RPD has led to high false positive rates and requires time-consuming manual screening. In addition to hazardous pathogens and toxins, current best practices are in place for chemical synthesis and distribution of controlled drugs (U.S. Department of Justice. Drug Enforcement Agency. Diversion Control Division, 2019) and chemical weapons (Headquarters Department of the Army, 2018), but bioengineering is enabling bioproduction of such materials (e.g., (Galanie et al., 2015; Nakagawa et al., 2016)), which may also require extra precaution for laboratory manipulation. Given the exponential rise in DNA synthesis orders (Vickers and Small, 2018) and widespread creation of biodesigns, current screening practices using traditional approaches are unsustainable due to the high cost burden (due to high labor costs associated with reviewing sequences) relative to the increasing low cost of nucleotide synthesis. Thus, the need exists to shift from a subjective, organism-centric to an objective (and cost-effective), function-centric biohazard identification and classification system. This need is at the forefront of best practices as new draft guidance for screening synthetic nucleotide orders opens the aperture for screening to “sequences of concern” from select and non-select agents from all nucleotide sequence types—including short sequences (Federal Registar, 2022).

Here we introduce the term “hazardous function,” which refers to one or more sequences (and associated metadata) that are associated with pathogenicity, toxicity, drug production, and other functions as described in this paper. Hazardous functions are driven by proteins that provide the organism or system (in the case of a cell free system or cell factory producing a toxin for example) with the necessary properties to cause infection or other detrimental effects. For example, lethal factor from *Bacillus anthracis* is a hazardous function, whereas DNA polymerase from *B. anthracis* is not. Manipulation of hazardous function sequences (e.g., recombinant production, genome insertion, mutation, etc.), even for legitimate purposes, could lead to the production of novel or enhanced hazardous products. In fact, precedent has shown that genetic manipulation can lead to biodesigns with high pathogenicity (van Der Most et al., 2000; Whitworth et al., 2005; Velmurugan et al., 2007; Bartra et al., 2008; Kurupati et al., 2010; Luo et al., 2010; Tsang et al., 2010), host bioregulation ability (Borzenkov et al., 1993; Borzenkov et al., 1994; Gold et al., 2007), vaccine escape capability (Serpinskii et al., 1996; Jackson et al., 2001; Zhang, 2003; Kerr et al., 2004; Chen et al., 2011), high transmissibility (Herfst et al., 2012), high toxicity (Francis et al., 2000), controlled drug production capability (Galanie et al., 2015; Nakagawa et al., 2016), and species extinction capability (Esvelt et al., 2014).

Hazardous functions identified through comparative genomic techniques (Gilmour et al., 2013) and related studies have been cataloged in databases containing virulence factors, toxins, and related other sequences (Supplementary Table S2). However, many of these databases are incomplete, poorly maintained, and/or do not have valuable metadata for objective biosafety assessments. Specifically, we and others have found that many of the entries in these databases simply tag sequences as “virulence factors” if attenuation of the activity leads to reduced virulence. Thus, many “virulence factors” may not be particularly hazardous in the context of bioengineering. For example, the Victor’s Virulence Factors Database (Sayers et al., 2019) compiles bacterial virulence factors implied from published experimentation, such as large-scale mutational screens that seek to identify attenuated virulence phenotypes. Niu et al. illustrated the controversy associated with the term “virulence factor” by determining that 69% (1,368/1,988) of virulence factors in the Virulence Factor Database (VFDB) (Liu et al., 2019a) were common among pathogens and non-pathogens (Niu et al., 2013). In a more specific example, Segura et al. calls into question the definition of “critical virulence factors” for *Streptococcus suis*, suggesting that more scrutiny is needed before characterizing a strain as virulent based on clinical presentation, animal models testing, or *in vitro* tests (Segura et al., 2017). Taken together, current databases do not serve the purpose needed for biohazard identification necessitating the need for better definition and curation around hazardous functions. Godbold et al. recently described a controlled vocabulary called Functions of Sequences of Concern microbial pathogenesis research for bioinformatic applications (Godbold GD et al., 2021). Here we demonstrate the utility of these types of sequences of concern for understanding biohazards associated with bioengineering functional building blocks.

Regardless of the controversy associated with the term *virulence factor*, it is clear that different functions (and context) have different levels of importance for determining the sequence’s overall hazard level and thus contribution to the organism or system’s hazard level. Given such wealth of publicly available knowledge on the functions derived from genetic sequences in UniProt (and related databases), databases such as those presented in Supplementary Table S2, and the scientific literature at large, the scientific community is primed to enable function-based DNA sequence assessment to aid in the preparation for novel pathogens and/or components with hazardous properties as well as prevent nefarious development of novel engineered pathogens. To anticipate potential hazards associated with novel pathogens, Colf et al. called for “functionality-based approach” that focuses on key hazard elements such as stability of an organism, infectious dose, or toxicity (Colf, 2016), but such practices have not fully come to fruition. Here we introduce a paradigm of function-based sequence assessment that may fill the gaps associated with current biosafety practices. Hazardous functions can be subjective based on what the user considers a “hazard,” but here we focus on functions associated with pathogenicity, toxicity, drug production, and other functions that can harm humans or other organisms of interest (e.g., livestock, crops, etc.). We first demonstrate our novel methodology to create a database of hazardous sequences classified into coarse functional categories. We then validate our methodology by demonstrating that a subset of the resulting database can be used to successfully distinguish pathogenic from nonpathogenic organisms via specific functional mechanisms. Finally, we further demonstrate the application of this methodology and resultant database through an example hazard scale. Therefore, the methodology demonstrated here can immediately be used for biosecurity screening assessments of synthetic genes (through the exemplar hazard scale) and partial biosafety assessments for classification of bacterial pathogens and non-pathogens. Because our methods rely on the DNA sequence’s encoded function, rather than agent-based lists, we provide a foundation for enabling function-based hazard assessments. This foundation can be built upon to provide comprehensive biosecurity and biosafety assessments for any novel biodesign through only analysis of the biodesign’s genome.

## Results

### A methodology and database for function-based hazard assessments

To enable function-based biohazard screening, we developed an access-controlled biological Functional Hazards Database that contains protein sequences with metadata. The database documents sequences that have been verified in the laboratory to encode a hazardous function based on experimental information from the primary literature and/or publicly available databases (e.g., Supplementary Table S2). We have compiled these sequences and metadata into a machine-readable database that is focused on biohazards that target humans and non-humans of high economic value. Non-human hosts are based on an analysis performed by the United States Department of Agriculture Economic Research that demonstrated cattle, poultry, and swine comprised 96% of U.S. livestock farm receipts (of $176 billion) and corn, soybeans, and wheat comprised 48% of U.S. crop farm receipts (of 195.4 billion) (United States Department of Agriculture Economic Research Service, 2022) in 2017. Together, these six commodities comprise 71% of all U.S. farm receipts in 2017 (United States Department of Agriculture Economic Research Service, 2022).

**FIGURE 2:**
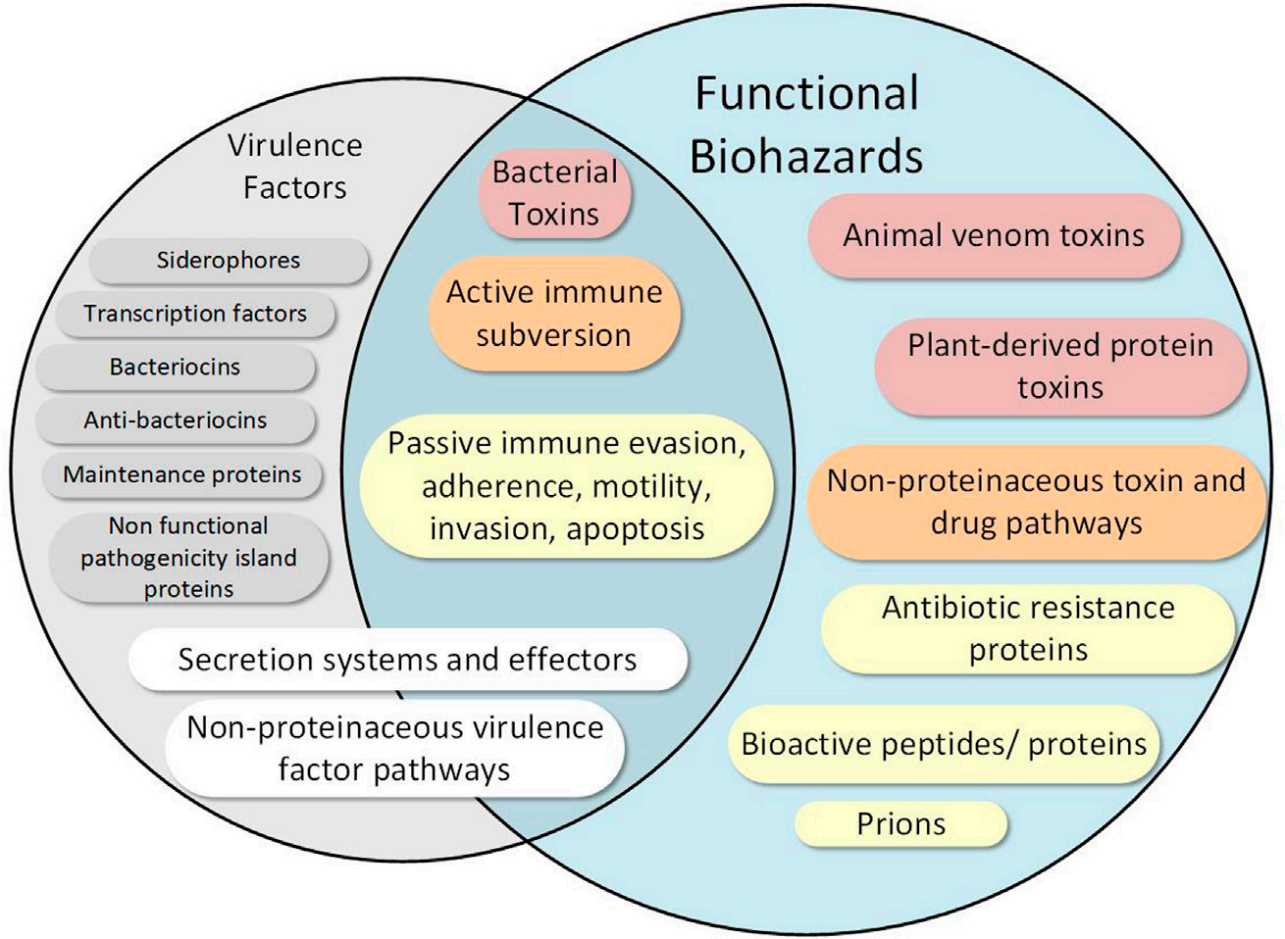
Functional biological hazards are differentiated from and include several functions beyond virulence factors. While some hazardous functions overlap with virulence factors, we define several hazardous functions outside the traditional definition of virulence factors. Many virulence factors that may be contained in avirulent organisms, such as siderophores and transcription factors, are not considered hazardous functions as they do not directly and uniquely perform hazardous functions. Hazardous functions are further described in the text and are color coded according coarse functional metadata groups as follows: Red—functions that do direct damage to cells such as toxins; Orange—functions involved in active host subversion or those involved in nonproteinacous toxin and drug pathways; Yellow—other virulence factors uniquely involved in pathogenicity (e.g., invasion), non-virulence factors that may contribute to detrimental host response (e.g., bioregulators and antibiotic resistance proteins), and prions; White—virulence factors that may also participate in non-hazardous microorganism functions; Gray—virulence factors that do not have a direct hazardous function. Note that the figure is non-exhaustive.

We focus our database on particularly hazardous functions, which includes only a subset of virulence factor types as well as several hazardous functions not considered virulence factors (Figure 2). We delineate a virulence factor from a hazardous function as follows: while a virulence factor describes any factor (protein or otherwise) that aids in the virulence of organism, we define functional hazards as any sequence whose *verified* encoded *function* can lead to a direct and harmful impact on a host given a biological vehicle to do so. Thus, a logical division between hazardous functions and virulence factors (Figure 2) emerges based on this definition. Some traditional virulence factors are thus considered hazards, such as those involved in evading the host’s immune system which–when encoded in an appropriate biological context (e.g., in *E. coli*)—contribute to direct detrimental impact to the host. In contrast, a transcription factor, for example, may only indirectly impact pathogenicity, and is thus not included in our hazard definition. We further delineate factors that are found throughout nature (i.e., those that are typically not unique to pathogens), such as siderophores, some secretion systems, and some non-protein virulence factor biosynthesis enzymes. For example, Type I and Type II secretion system proteins, which are ubiquitous throughout all gram-negative bacteria—pathogens and non-pathogens (Green and Mecsas, 2016)—are not considered hazardous functions in our definition. In contrast, Types III and IV secretion system proteins, which enable transport of potentially hazardous payloads across two gram-negative bacterial membranes and a host membrane, are considered hazardous functions. Further, careful consideration is given to particularly hazardous non-protein virulence factors such as endotoxin, which is biosynthesized by several enzymes (Raetz and Whitfield, 2002). More importantly, we consider several other sequence types that are not considered traditional virulence factors to be hazardous functions, such as prions, bioregulators, animal toxins (e.g., conotoxins), protein toxins (e.g., ricin), and proteins involved in the biosynthesis of small molecule toxins (e.g., saxitoxin) and drugs (e.g., morphine). For all hazardous sequences, we functionally classify the type of hazardous function into one or more of the 15 high level categories in Table 1 and elaborated below. These categories, chosen based on previous expert discussions from scientists with a variety of life science backgrounds, provide the basis for distinguishing pathogens and nonpathogens as shown by our validation and example biosafety assessment hazard scale discussed later.

**TABLE 1:** Hazardous functional metadata categories.

Functional metadata	Definition
Adherence	Mediates pathogen or toxin binding to host cell
Motility	Enables a pathogen to move within or between host cells
Invasion	Enables a pathogen or toxin to actively enter or maintain protected spaces within the host
Inhibits host cell death	Inhibits host cell death
Host cell apoptosis	Leads to, aids in, and/or promotes host cell death
Passive host subversion	Passively works to avoid the immune surveillance, e.g., by altering recognizable elements of the pathogen
Active host subversion	Actively aggravates host immune detectors or effectors
Antibiotic resistance	Enables resistance of a pathogen to antibiotics
Damage	Actively damages host cells, host cell processes, or host barriers such as the extracellular matrix. Toxin sequences specifically contain the toxin activity gene ontology term (GO: GO:0009636)
Toxin pathway	Directly involved in the biosynthesis of a non-proteinaceous toxin
Drug pathway	Directly involved in the biosynthesis of a non-proteinaceous drug
Protein Bioregulators	Regulates cellular processes that can be detrimental to the host
Bioregulator pathway	Directly involved in the biosynthesis of a non-proteinaceous bioregulator that can be detrimental to the host
Prion	Protein that can misfold to become an infectious agent
Unknown	Hazardous function is unknown but contributes to complete or near complete loss of virulence when deleted or mutated

#### Adherence, invasion, and motility

Adherence factors contained within our functional hazard database have experimental evidence (e.g., immunoprecipitation, cell binding assay, etc.) of a direct interaction with host membrane components. Interaction between the adherence factor and the host may enhance host cell tropism through direct interactions of a pathogenic apparatus that binds surface host cell receptors. Proteins that do not directly interact with the host but may be required for assembly of such a pathogenic apparatus can also be considered adherence factors but are further identified in our database as being dependent on direct adherence factors. For example, a type-4 pilus apparatus is responsible for adherence of *Neisseria meningitidis* to host receptors (Rudel et al., 1995), but is composed of several protein subunits. PilC and PilE have direct interactions with the host, whereas other proteins in the assembly do not (Bernard et al., 2014).

Invasion factors are those that leverage mechanisms such as Type III or Type IV Secretion Systems (T3SS/T4SS), pore formation, actin polymerization dysregulation, or cell lysis. The T3SS is a multi-protein needle complex that allows bacterial effectors to be delivered from the pathogen into the host cell directly. These effector proteins promote infection and suppress host defenses. For example, the *Yersinia pestis* T3SS structure includes nearly 40 proteins (Cornelis, 2000; Frolkis et al., 2010). In *Y. pestis*, T3SS activation is triggered by cell contact and induces the secretion of effector proteins—termed Yersinia outer proteins (Yops)—across the host cell membrane where they inhibit bacterial phagocytosis and suppress the host immune response (Plano and Schesser, 2013). Like T3SSs, sequences such as bacterial pore-forming lysins and fungal cutinases, which can enable invasion through cleaving host cell walls (Sweigard et al., 1992; Dean et al., 2005; Chen et al., 2007; Basso et al., 2017) are included as well. Other types of invasive bacterial proteins, such as invasion plasmid antigen A (IpaA) from *Shigella sp*., which enables invasion through actin dysregulation (Izard et al., 2006; Park et al., 2011), are also included.

In addition to adherence and invasion, we include some motility factors, as some pathogens use mechanisms that allow a microbe to actively move between or within host cells following infection. This phenomenon is known as actin-based motility, which involves subversion of the host actin cytoskeleton to stimulate movement within the host cell, ultimately leading to microbial spread between cells. This rapid microbial dissemination is a critical step in many infectious diseases. For example, diseases caused by *Listeria monocytogenes* are caused in part by the protein ActA, which directly activates host actin polymerization machinery. This activation results in the formation of an actin “rocket tail” that propels the bacteria into adjacent cells, thereby infecting them (Finlay, 2005; Ireton, 2013).

#### Host cell death

During infection, pathogens work to maintain tight control of the host’s intrinsic cell death mechanisms, often suppressing cell death then activating it to allow replication then dissemination, respectively. Induction of host cell death is used as a pathogenic strategy to allow a virus or bacteria to efficiently exit the host cell, spread to neighboring cells and access nutrients (Ashida et al., 2011). Further, by inducing host cell death, a pathogen can also eliminate immune cells and effectively evade immune defenses (Lamkanfi and Dixit, 2010; Ashida et al., 2011). Viruses are common proponents of this mechanism to facilitate dissemination of replicated virus and suppression of the immune system. For example, the human immunodeficiency virus (HIV), induces programed cell death in healthy T lymphocytes, contributing to the gradual T cell decline and ultimately acquired immune deficiency syndrome (Ahr et al., 2004; Romani and Engelbrecht, 2009). Thus, proteins such those that promote this induction of apoptotic signal (Vpr and HIV envelope proteins) are including in our database (Ayyavoo et al., 1997; Ahr et al., 2004; Romani and Engelbrecht, 2009). In contrast to induction of host cell death, inhibition of host cell death is also a hazardous function since host cell death can be used as an immune defense mechanism to contain the spread of the infection. These hazardous functions enable a pathogen to promote its overall survival within the host by giving the pathogen more time to colonize efficiently prior to dissemination. Enteropathogenic *Escherichia coli*, for example, uses this strategy to stall premature host cell death during infection through the EspZ effector protein, which activates pro-survival signaling pathways within the host (Shames et al., 2010; Shames and Finlay, 2010).

#### Passive and active host subversion

Pathogens can also evade the host by avoiding or aggregating more specific host immune defenses than those discussed above. Microbes have evolved numerous and diverse strategies to circumvent the host immune system, many even using multiple mechanisms. We classify these strategies as passive or active, in which hazardous functions act to either avoid host immune surveillance or actively interfere with the host’s immune responses, respectively. Common passive mechanisms include using antigenic variation, epitope masking, and the use of decoys or molecular mimicry. Often, circumvention of host detection is accomplished by a virulence factor altering recognizable elements of the pathogen. For example, Ebola virus glycoprotein (GP), a key antigen in Ebola pathogenesis, can evade host immune defenses by epitope masking and steric shielding (Cook and Lee, 2013; Wong et al., 2014). Steric shielding of surface epitopes by glycans also prevents antibody binding and binding of host major histone compatible complex I and β1 integrins with other immune cells, thereby preventing the host immune response (Francica et al., 2010). Ebola virus also leverages decoy mechanisms by producing large quantities of secreted GP proteins that adsorb host antibodies (Blair et al., 2015).

In contrast to passive subversion, active host subversion involves active interference with the host’s immune responses. For such interference, a microbe must produce factors that are able to block or modulate specific steps in the immune response cascade (Schmid-Hempel, 2009). These factors can be membrane-bound or directly injected directly into the host cell using secretion systems such T3SSs, as discussed above (Raymond et al., 2013). Many bacteria possess efficient means of evading the host complement system. For example, chemotaxis inhibitory protein (CHIPS) from *S. aureus* can bind receptors on neutrophils, blocking their recruitment and engagement to resist complement-mediated killing (Rooijakkers et al., 2005). Active evasion of the immune system can also be accomplished by interfering with the immune response signaling network. For example, *Yersinia* Yop proteins downregulate the expression of TNF-α, thereby effectively blocking pro-inflammatory signaling (Sweet et al., 2007; Schmid-Hempel, 2009).

#### Antibiotic resistance

Just as pathogens can evade endogenous host responses, pathogens have evolved to evade exogenous factors, such as antibiotics, through expressing hazardous functions. Surveillance of these hazardous functions is critical, as the rapid and broad dissemination of antibiotic resistance determinants by lateral gene transfer has been demonstrated throughout diverse bacterial species. Several mechanisms have been described that can lead to antibiotic resistance including: production of enzymes capable of metabolizing or modifying antibiotics, antibiotic binding-site modifications to prevent binding, production of outer membrane components that confer low permeability, and overexpression of multi-drug efflux pumps (Fournier et al., 2006; Vila et al., 2007; Kempf and Rolain, 2012; Blair et al., 2015; Bakour et al., 2016; Geisinger and Isberg, 2017). Bacteria often employ more than one mechanism of antibiotic resistance, leading to multidrug-resistant strains. For example, methicillin resistant *S. aureus* (MRSA), produce both β-lactamases that can inactivate β-lactam antibiotics (e.g., penicillin), as well as proteins acquired by lateral gene transfer (PBP2a proteins) that confer resistance to methicillin (Chambers, 1997; Stapleton and Taylor, 2002). While antibiotic resistance factors can be hazardous, the context of the factors needs to be carefully considered. Often antibiotic resistance has been shown to result in virulence attenuation (Andersson and Hughes, 2010; Geisinger and Isberg, 2017), but some studies demonstrate that resistance has increased pathogenic potential during infection (Luo et al., 2005; Skurnik et al., 2013; Roux et al., 2015). While the precise correlation between virulence and antibiotic resistance remains unclear, we define antibiotic resistance as hazardous function given reasonable context (i.e., contained within a pathogen).

#### Damage

Perhaps the most hazardous functional category can be considered one that does direct damage to the host. While some of the above hazardous functions can directly damage the host, biological toxins represent the largest class of directly damaging hazardous functions. According to the Gene Ontology Consortium, biological toxin activity involves the selective interaction “with one or more biological molecules in another organism (the “target” organism), initiating pathogenesis (leading to an abnormal, generally detrimental state) in the target organism” (EMBL-EBI, 2019). Biological toxins may be proteinaceous or non-proteinaceous, with protein toxins often consisting of multiple subunits that attribute to virulent functions for adherence, invasion, and inactivation of critical cellular functions. Toxins are highly diverse, even within some toxin types. For example, possibly hundreds of thousands of conotoxins—antagonists or agonists of various receptors and ion channels—exist (Lewis et al., 2012). Examples of proteins relevant to this category included in our hazardous function database are shown in Supplementary Table S4.

#### Pathways

In addition to protein toxins, our database includes key enzymes involved in the biosynthesis of fully and partially characterized small molecule toxin pathways, such as those that produce aflatoxins (cancer-causing and cellular process-disruption fungal toxins (Haschek and Voss, 2013; National Cancer Institute, 2019)), trichothecenes mycotoxins (protein synthesis-inhibiting fungal toxins (Kiessling, 1986)), microcystins (cyanobacterial serine/threonine protein phosphatase-hepatotoxins (Tillett et al., 2000; Campos and Vasconcelos, 2010)), tetrodotoxins (bacterial sodium channel-blocking neurotoxins) (Jal and Khora, 2015; Lago et al., 2015; Magarlamov et al., 2017), and saxitoxins (bacterial sodium channel-blocking neurotoxins) (Al-Tebrineh et al., 2010).

Beyond hazardous pathogens and toxins, we also consider naturally derived or inspired drugs. Bioengineering is presenting a new challenge to control the production of these naturally derived drugs, as the starting materials may not be regulated. Some drugs, such as opiates and cannabinoids, are produced naturally in plants, and have been demonstrated to be produced in yeast and bacteria (Galanie et al., 2015; Poulos and Farnia, 2015; Nakagawa et al., 2016). Illicit drugs pose a hazard to public health and the economy and are thus controlled by the US Drug Enforcement Administration (DEA) using a five category classification system (United States Drug Enforcement Administration, 2019), with schedule I drugs being the highest hazards as they have no currently accepted medical use and have a high potential for abuse (e.g., heroin and cannabis). For chemical synthesis, supplies to synthesize drugs are regulated by the US government (U.S. Department of Justice. Drug Enforcement Agency. Diversion Control Division, 2019), but biosynthetic supplies are less regulated and may thus present a gap in biosecurity and biosafety. Our functional hazards database thus includes exemplar pathways such as the opioid and cannabinoid pathways, which are fairly well elucidated (Galanie et al., 2015; Nakagawa et al., 2016) as well as sequences from less characterized pathways, such as the cocaine pathway (Jirschitzka et al., 2012).

#### Bioregulators

We also consider host regulators as well, since such molecules can ultimately lead to manifestations of disease (Goldman, 2000) and have drug-like activity. These bioregulators can be peptides, proteins, and small molecules produced naturally by the host in response to an insult or produced by other organisms (e.g., amphibians). Further, regulatory peptides have been discovered and created to mimic small molecule regulators such as opioids (Dudak et al., 2011; Aldrich and McLaughlin, 2012). Like antibiotic resistance factors, the context and scope of bioregulators must be carefully considered. While many bioregulators can be considered hazardous, we limited our initial database to those that could have a high impact on human systems such as the cardiovascular, nervous, and immune systems (Supplementary Table S3).

#### Prions

Prions are considered a functional hazard as well. A prion is a protein that can misfold to become an infectious agent (i.e., transmitted from one host to another). Prions most abundantly occur in the brain and are responsible for a variety of fatal progressive neurodegenerative disorders called transmissible spongiform encephalopathies (Prusiner, 1998). The causative agents of these diseases are normal cellular prion proteins (PrPC) that have undergone a posttranslational conformational change into an abnormal scrapie prion protein (PrPSc) (Huang et al., 2015). PrPSc proteins are able to transmit the pathological conformation to PrPC through poorly understood mechanisms (Dobson, 2001; Huang et al., 2015; Erana and Castilla, 2016). Notable prions included in our database are those that lead to Bovine Spongiform Encephalopothy (BSE, or “mad cow disease”), Creutzfeldt-Jakob disease in humans, feline spongiform encephalopathy in cats, and exotic ungulate encephalopathy in zoo animals (Wells et al., 1987; Wilesmith, 1994; Will et al., 1996). Although these diseases are rare, they are usually rapidly progressive and fatal and synthetic versions can induce pathology in experimental animals (Telling et al., 1995; Legname et al., 2004).

#### Unknown

While many hazardous functions have distinct mechanisms, we do consider potentially hazardous functions with nonspecific mechanisms as well. Throughout the database compilation process, we identified several instances where a protein sequence likely contributes to a hazardous function, but the exact mechanism is unknown. For example, our database contains a relatively high number of Mycobacterium sequences since we leveraged many of the virulence factors documented in PATRIC (Wattam et al., 2017), which relied mainly on one study. In this study, the authors identified which genes are required for *in vivo* growth (and not *in vitro* growth) (Sassetti and Rubin, 2003). Thus, while many of these genes are considered to potentially contribute to hazardous functions, their actual functions are unknown.

### Validation of the methodology and resulting functional hazard database: Identification of hazardous functions

To validate our methodology of identifying, categorizing, and databasing hazardous sequences, we leveraged the studies presented in Table 3, which segregate various pathogenic and nonpathogenic bacterial species. We identified eight different organism groups and separated species in each group into pathogens and nonpathogens. We further categorize the pathogens into species and/or disease-causing groups. With the exception of *Pseudomonas syringae* (a plant pathogen), all species leveraged in this validation are pathogenic to humans and/or economically critical livestock. For the validation, we aligned the coding sequences (CDSs) from each strain against a subset of our database that contained only hazardous function sequences from each of the eight organism groups. We used a subset of our database to reduce potential noise associated with hazardous functions potentially encoded in nonpathogens as a proof of concept for the method; thus any use of this methodology for biosafety assessments should note this limitation. We scored each CDS alignment hit as the *(percent identity) × (percent hazardous sequence coverage)* and normalized each hit to the total number of CDSs contained in the strain. The normalization step was performed since, for example in the case of *E. coli*, 1 Mb genome size differences can occur among strains, leading to different pathotypes (Dobrindt, 2005). To count the fraction of hazardous CDSs in each strain, we considered different alignment thresholds to ensure that a specific alignment cutoff did not impact our results. Specifically, the fraction of hazardous sequences is nearly unchanged between 20 and 80% alignment scores for all groups (data not shown). Importantly, the fraction of hazardous functions in pathogenic species compared to nonpathogenic species is higher across the entire range in nearly all cases.

Table 2 shows the number and percentage of hazardous CDSs using a relatively stringent alignment threshold of 40%. The 40% threshold has previously been demonstrated to be a useful cutoff by Suzek et al. (2015). In the referenced study, the authors showed 97% of Uniref50 cluster members, defined by the 40% threshold (≥50% sequence identity over 80% sequence coverage (UniProt, 2019a)), share identical or similar gene ontology terms (i.e., have the same function) (Suzek et al., 2015). Thus, this threshold is useful for CDSs that have identical or similar functions relative to sequences contained in the hazardous function database. Table 3 outlines that the average number and fraction of CDSs identified for each pathogenic and nonpathogenic group using the 40% threshold. In 19 out of 21 pathogenic groups, the percentage of CDSs is higher for pathogens compared to nonpathogens (16/18 being significantly higher), suggesting that our methodology was successful in identifying hazardous functions for these groups.

**TABLE 2:** The average number and percentage of hazardous CDSs are greater in pathogenic groups compared to nonpathogenic Groups.

Organism	Group	Genera/species in group	Average ± SD of # CDSs with hazardous functions (Average % CDSs)^a^
Neisseria	Pathogenic	*N. meningitidis*	***50 ± 3 (2.3%)***
		*N. gonorrhoeae*	***53 ± 3 (2.2%)***
	Nonpathogenic	See Table 3	27 ± 5 (1.3%)
*Escherichia coli*	Pathogenic	EAEC/ETEC/AIEC/EPEC	160 ± 31 (3.2%)
		EHEC	***290 ± 20 (5.3%)***
		ExPEC	***163 ± 32 (3.3%)***
	Nonpathogenic	See Table 3	125 ± 26 (2.7%)
Burkholderia	Pathogenic	*B. mallei*	***111 ± 17 (2.1%)***
		*B. pseudomallei*	***143 ± 16 (2.1%)***
		*B. cenocepacia*	***102 ± 8 (1.5%)***
	Nonpathogenic	See Table 3	82 ± 20 (1.2%)
Pseudomonas	Pathogenic	*P. aeruginosa* and *P. mendocina*	***126 ± 21 (2.3%)***
		*P. syringae* (plant pathogen)	76 ± 3 (1.3%)
	Nonpathogenic	See Table 3	76 ± 7 (1.9%)
Streptococcus	Pathogenic	*S. pneumoniae*	***39 ± 6 (1.9%)***
		*S. pyogenes*	***42 ± 3 (2.2%)***
		*S. suis*	***33 ± 4 (1.6%)***
	Nonpathogenic	See Table 3	20 ± 2 (1.0%)
Bacillus	Pathogenic	*B. cereus* and others (See Table 3)	***59 ± 11 (1.1%)***
		*B. anthracis*	***61 ± 3 (1.1%)***
	Nonpathogenic	See Table 3	23 ± 10 (0.5%)
Clostridium	Pathogenic	*C. botulinum* and *C. tetani*	***6 ± 1 (0.3%)***
		*C. difficile*	5 ± 1 (0.2%)
		*C. perfringens*	***5 ± 1 (0.4%)***
	Nonpathogenic	See Table 3	1 ± 1 (0.1%)
Mycobacterium	Pathogenic	*M. tuberculosis* and others (See Table 3)	***440 ± 8 (26%)***
		*M. leprae* and others (See Table 3)	***281 ± 120 (18%)***
	Nonpathogenic	See Table 3	288 ± 19 (12%)

a CDSs above the 40% threshold as defined in the Methods Section; the fraction of CDSs is defined by the number of hits divided by the total number of CDSs in each strain.

***Bold italics*** represents a significant difference in percentage between the pathogenic and nonpathogenic group as defined by a pairwise *t*-test (*p* < 0.05, two-tailed, unequal variance).

**TABLE 3:** Genomic data from pathogenic and nonpathogenic strains used in this study.

Type	Genera/Species organism group	References	Pathogenic groups: species/strains (#)	Nonpathogenic groups: species/strains (#)	# Hazardous functions in database
Gram-negative bacteria	Neisseria	Lu et al. (2019)	*1. N. meningitidis* (85)	*N. lactamica* (3); *N. longa* (1); *N. zoodegmatis* (1); *N. longate* (1)	67
			*2. N. gonorrhoeae* (15)		
Gram-negative bacteria	*Escherichia coli*	Cosentino et al. (2013)	1. EAEC/ETEC/AIEC/EPEC (11)	K-12 (2); other non-pathogenic strains (13)	374
			2. EHEC (8)		
			3. ExPEC (10)		
Gram-negative bacteria	Burkholderia	Cosentino et al. (2013)	*1. B. mallei* (4)	*B.* sp. *CCGE1001* (1); *B.* sp. *YI23* (1); *B. glumae BGR1* (1); B. *phymatum STM815* (1); *B. phytofirmans PsJN* (1)	141
			*2. B. pseudomallei* (4)		
			*3. B. cenocepacia* (4)		
Gram-negative bacteria	Pseudomonas	Cosentino et al. (2013)	*1. P. aeruginosa* (5) and *P. mendocina* (2)	*P. brassicacearum* (1); *P. fluorescens* (2); *P. putida* (6); *P. stutzeri* (1)	175
			*2. P. syringae* (3)		
Gram-positive bacteria	Streptococcus	Cosentino et al. (2013)	*1. S. pneumoniae* (9)	*S. parauberis* (1); *S. salivarius* (3); *S. thermophilus* (5)	161
			*2. S. pyogenes* (13)		
			*3. S. suis* (9)		
Gram-positive bacteria	Bacillus	Cosentino et al. (2013)	*1. B. cereus* (6); *B. cytotoxicus* (1); *B. weihenstephanensis* (1)	*B. amyloliquefaciens* (4); *B. atrophaeus* (1); *B. cellulosilyticus* (1); *B. cereus Q1* (1); *B. clausii* (1); *B. coagulans* (2); *B. halodurans* (1); *B. megaterium* (1)	116
			*2. B. anthracis* (5)	*B. pumilus* (1); *B. selenitireducens* (1); *B. subtilis* (4)	
Gram-positive bacteria	Clostridium	Cosentino et al. (2013)	*1. C. botulinum* (8) and *C. tetani* (1)	*C. acetobutylicum* (3); *C. beijerinckii* (1); *C. cellulovorans* (1); *C. clariflavum* (1); *C. kluyveri* (2); *C. lentocellum* (1); *C. ljungdahlii* (1); *C. phytofermentans* (1); *C. saccharolyticum* (1); *C.* sp. *SY8519* (1); *C. thermocellum* (1)	54
			*2. C. difficile* (2)		
			*3. C. perfringens* (3)		
Bacteria	Mycobacterium	Andreevskaia et al. (2006); Cosentino et al. (2013); Ilina et al. (2013); Prasanna and Mehra (2013)	*1. M. africanum* (1); *M. avium* (1); *M. bovis* (1); *M. canettii* (1); *M. tuberculosis* (5)	*M.* sp. *KMS* (1); *M. gilvum* (1)	339
			*2. M. abscessus* (1); *M. avium* (1); *M. leprae* (2); *M. marinum* (1); *M. ulcerans* (1)	*M. rhodesiae* (1); *M. smegmatis* (1); *M.* sp. *JLS* (1); *M.* sp. *MCS* (1)	
				*M.* sp. *Spyr1* (1); *M. vanbaalenii* (1)	

We further identified specific hazardous functions enriched in each pathogenic group (Supplementary Table S1). For this analysis, we assumed (based on testing, data not shown) that a function is “enriched” in a pathogenic group compared to its nonpathogenic counterpart if the average alignment score across all strains in the group is ≥60% higher than the average in the nonpathogen group or the average in the nonpathogen group is 0% and the average in the pathogen group is ≥40%. As a control, we also determined if any hazardous functions are enriched in the nonpathogen group (i.e., if the average alignment score in the nonpathogenic group is ≥60% higher than the pathogen group or the average in the pathogen group is 0% and the average in the nonpathogen group is ≥40%). Based on this analysis, we identified 379 total enriched functions in the pathogenic groups compared to only 12 total hazardous functions in the nonpathogen groups. The pathogen groups averaged 19 enriched hazardous functions across the various pathogen groups (range 1–70, Supplementary Table S1). These functions were involved in a variety of processes such as adherence, immune evasion, antibiotic resistance and damage (including toxin activity). The hazardous functions identified to be enriched in the nonpathogen groups mapped to four functions in the *E. coli* group (required for colonization but with unknown mechanisms), one antibiotic resitance function in the *P. syringae* group, three functions in the in the *S. pyogenes* group (involved in antiphagocytosis but with unknown mechanisms), and four antibiotic resistance functions in the Mycobacterium groups. Thus, the results in Supplementary Table S1 suggest that our database enables successful identification of enriched hazardous functions from pathogens as compared to their nonpathogenic counterparts.

### Validation of the methodology and resulting functional hazard database: Hazard fingerprints

**FIGURE 3:**
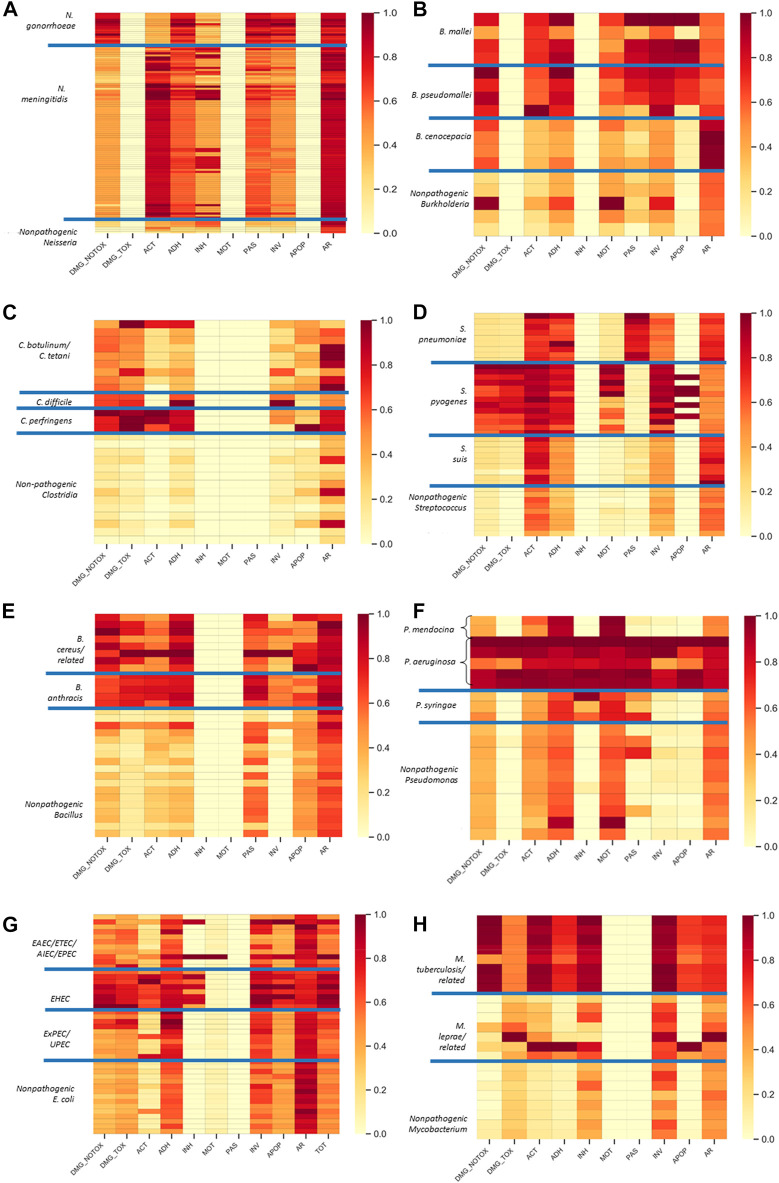
Pathogenic species are enriched in hazardous functional categories. Shown are the hazard fingerprints for Neisseria **(A)**, Burkholderia **(B)**, Clostridium **(C)**, Streptococcus **(D)**, Bacillus **(E)**, Pseudomonas **(F)**, *E. coli* **(G)**, and Mycobacterium **(H)**. The fingerprints are shown as rows in a heat plot with the values in each column representing the normalized fraction of CDSs within each functional category (as defined in Table 1). Only the relevant categories from Table 1 are included (i.e., those that provided alignments). The pathogenic subgroups within each organism group are defined in Table 3 and separated by the blue lines on the heat plot. Abbreviations: DMG_NOTOX, damage without toxin activity GO term, DMG_TOX, damage with toxin activity GO term; ACT, active host subversion; ADH, adherence; INH, inhibits host cell death; MOT, motility; PAS, passive host subversion; INV, invasion; APOP, host cell apoptosis; AR, antibiotic resistance; TOT, TOTAL (sum of all other categories).

**FIGURE 4:**
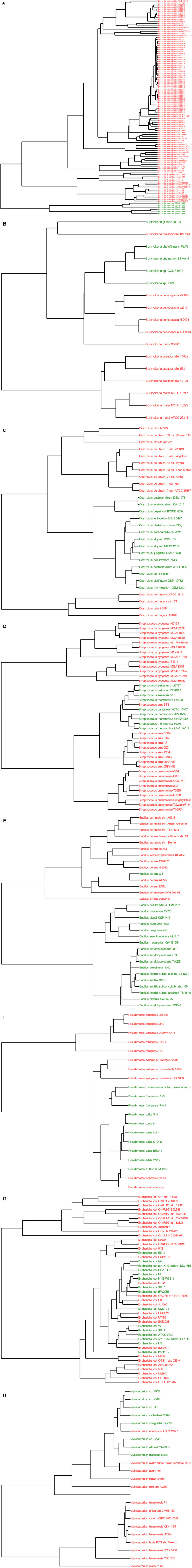
Hazardous functions separate pathogens from non-pathogens. Shown are the dendrograms for Neisseria **(A)**, Burkholderia **(B)**, Clostridium **(C)**, Streptococcus **(D)**, Bacillus **(E)**, Pseudomonas **(F)**, *E. coli* **(G)**, and Mycobacterium **(H)**, with pathogenic species colored in red and non-pathogens colored in green. An additional plot for *E. coli*, stratified by the groups shown in Figure 3 is shown in Supplementary Figure S1.

To validate the classification component of our methodology (Table 1), we leveraged our functional categories to create “hazard fingerprints” for each strain. The fingerprints were calculated by summing the alignment scores for the CDSs for each strain that belong to each functional category. For these alignments, we accounted for both highly confident hazardous CDSs (e.g., those with alignment scores >40% to our database) as well as less confident, yet potentially hazardous functions by summing all qualified alignment scores as described in the Methods section. This approach allows for more score contribution for higher identity alignments while still allowing for some contribution for lower identity alignments. We then normalized the scores within each functional category by dividing each value by the maximum value in that functional category. This normalization enables critical hazardous functions that may only be encoded with one or a few CDSs (e.g., a critical toxin) that are absent in nonpathogens to be emphasized within a category and controls for abundance bias within our hazard database across functional categories. For this analysis, we considered only known functions (i.e., the “unknown” functional category Table 1 was excluded) to remove noise from the analysis stemming from sequences with potentially hazardous but unknown functionalities. Figure 3 shows the fingerprints for each of the eight organism groups in the form of heat plots to study visual differences among the various hazard categories. We further analyzed the hazard fingerprint data from the heat plots using agglomerative hierarchical cluster analysis. These clusters were then visualized by plotting dendrograms, where known pathogenic groups were labeled in red, and non-pathogenic in green. For most organisms, hierarchical clustering based on the fingerprint data effectively distinguished between pathogenic and non-pathogenic strains Figure 4).

Overall, the plots demonstrate high levels of hazardous functions in pathogens relative to nonpathogens (Figure 3) and good separation between pathogen and non pathogens (Figure 4). More specifically for the fingerprints, there is good separation across most categories with the exception of antibiotic resistance, and the types of hazardous functions are consistent with literature reports as described below. For example, as shown in Figure 3A, both pathogenic Neisseria groups are enriched relative to the nonpathogen group in adherence, passive host subversion, and invasion functions. Further, the dendrogram demonstrates clear separation between pathogens and nonpathogens (Figure 4A). These findings are consistent with Lu et al., who demonstrated several genes unique to pathogenic Neisseria species that are involved in host immune evasion and adherence (Lu et al., 2019). *N. gonorrhoeae* further contains strains enriched in critical non-toxin damage functions, and *N. meningitidis* is enriched in active host subversion functions such Factor H binding protein (Supplementary Table S1).

Similarly, pathogenic Clostridium groups are clearly separated (Figure 4C), and pathogens are enriched in damage, adherence, and invasion functions relative to the nonpathogen group, with some strains being enriched in active host subversion and apoptosis (particularly the *C. perfringens* group) (Figure 3C). The most striking of these enriched categories for Clostridium are the damage categories, which is consistent with various Clostridium species producing damage-inducing factors such as toxins as their main hazardous functions, of which some can aggravate the immune response (Supplementary Table S1). For example, *C. botulinum* produces neurotoxins, *C. difficile* produces toxin A, toxin B, and binary toxin, and *C. perfringens* produces over 16 toxins (Awad et al., 2014; Rasool et al., 2017). Because the numbers of toxins produced by *C. perfringens* relative to the other two pathogenic groups is relatively higher compared to the other pathogenic groups, greater delineation between this pathogen group and the nonpathogenic Clostridium group is apparent due to the normalization process.

The Bacillus fingerprints (Figure 3E) demonstrates that Bacillus pathogens are enriched in functions related to damage, active host subversion and adherence relative to their nonpathogenic groups. The fingerprint plot also demonstrates that nonpathogenic Bacillus have antibiotic resistance functions, which supports other reports (Adimpong et al., 2012; Noor Uddin et al., 2015). For *B. anthracis*, the damage and active host subversion are most clearly delineated from the nonpathogen group, which is consistent with anthrax toxin—composed of protective antigen, edema factor and lethal factor (Supplementary Table S1)—being the major contributor to disease through destruction of host immune cells (Friebe et al., 2016; Visiello et al., 2016). Similarly, *B. cereus* contains factors that promote cell (including immune cell) damage, such as enterotoxins, hemolysins, emetic toxins, and phospholipases (Supplementary Table S1) (Visiello et al., 2016). Taken together, these functions allow separation of pathogens and non-pathogens (Figure 4E), with exception of one presumably non-pathogenic B. cereus strain Q1, an extremophilic strain known for microbial enhanced oil recovery due to production of biosurfactants (Xiong et al., 2009).

The plots also show good separation of some of the Streptococcus species from the nonpathogenic groups, particularly *S. pyogenes* (Figures 3, 4D). *S. pyogenes*—known as Group A Streptococcus clinically—has several factors enabling invasion, adherence, and motility within host cells, but perhaps the most important factors contributing to pathogenicity of *S. pyogenes* are the few proteins leading to direct damage (e.g., streptolysins O and S, and exotoxins A and C) and host evasion (e.g., IgG-degrading enzyme and Protein M) (Hamada et al., 2015). These critical functions are apparent in the heat plot as well as Supplementary Table S1. Less defined separation is apparent between the nonpathogenic group and the *S. pneumoniae* or *S. suis* group with a few exceptions. For example, antibiotic resistance factors show some delineation from the nonpathogen and *S. pneumoniae* or *S. suis* groups, which is consistent with the emergence of antibiotic resistance strains in these species (Nuermberger and Bishai, 2004; Yongkiettrakul et al., 2019). Further, enzymes leading to *S. pneumoniae* cell wall decoration that enable immune system avoidance (Mitchell and Mitchell, 2010) likely contributes to this group being separated from the other groups within the passive immune subversion category. *S. pneumoniae* and *S. suis* also express critical damage factors, such as the PLY pore-forming toxin (Mitchell and Mitchell, 2010) and hemolysins (Haas and Grenier, 2018), respectively, which—while not very apparent in Figure 3 due to high levels of the damage functional category in *S. pyogenes*—are identified as critical factors in Supplementary Table S1. Taken together, these hazardous functions enable good separation of pathogens from non pathogens. One exception is the pathogenic strain *S. suis ST3*. According to this Hu et al., this strain is missing a large pathogenicity island (Hu et al., 2011), which is the likely cause of lack of separation.

Like Streptococcus pathogens, Mycobacterium pathogens, particularly tuberculosis-causing Mycobacteria, are separated well within specific hazardous categories (Figure 3H) and separate well from non-pathogens (Figure 4H). One exception is *M. abscessus ATCC 19977*, a pathogen that clusters with non pathogens. This finding is actually consistent with another report, which demonstrated that this strain clusters with other non-pathogens based on whole proteome analysis (Zakham et al., 2012). In general, we found that *M. tuberculosis* strains are enriched in active host subversion, adherence, and apoptosis categories relative to the nonpathogen group, which is consistent with the fact that *M. tuberculosis* virulence largely depends on the organism’s ability to infect host cells and evade the host immune response (Forrellad et al., 2013). The plot additionally shows that damage factors contribute to differences compared to the nonpathogen group, which supports the fact that *M. tuberculosis* requires damage factors such as adenylate cyclase (Supplementary Table S1) for virulence (Agarwal et al., 2009). In contrast to *M. tuberculosis*, less separation is apparent for the *M. leprae* and related group. This observation is likely because only 24 of the 339 Mycobacterium hazardous functions contained in our database are from the *M. leprae* and related group, and the CDSs from this group may not have enough homology to hazardous functions from *M. tuberculosis* strains to be relevant in our analysis.

Similar to the Mycobacterium analyses, some hazardous categories are emphasized for *E. coli*, although our analysis was not able to clearly separate all pathogenic groups (Note: Figure 4G colors and labels the dendrogram based on pathogenic and non-pathogenic strains, whereas Supplementary Figure S1 colors by pathogenic and non-pathogenic group). Since infections caused from intestinal pathogenic *E. coli* (IPEC) are distinct from infections caused extraintestinal pathogenic *E. coli* (ExPEC, including uropathogenic *E. coli*) (Kohler and Dobrindt, 2011), we separated with *E. coli* pathogenic strains into IPEC strains—including a group of enterohaemorrhagic *E. coli* (EHEC) and non-EHEC strains (EAEC/ETEC/AIEC/EPEC)—and ExPEC strains. While EHEC strains are clearly separated (Supplementary Figure S1), ExPEC strains could not be separated as well, likely because these strains can belong to the normal (nonpathogenic) gut flora and share large portions of their genome with nonpathogenic strains (Kohler and Dobrindt, 2011). In contrast to the ExPEC strains, the IPEC strains—particularly the EHEC strains—show greater relative abundance of damage functions (Figure 3E). This observation supports that fact that functions that contribute to host cell damage are critical to IPEC pathogenesis, such as enterotoxins and shigatoxins (within ETEC and EHEC strains, respectively) as well as functions leading to attaching and effacing lesions (Welch et al., 2002; Kaur et al., 2010; Nguyen and Sperandio, 2012). The EHEC group is also further differentiated from the other IPEC strains within the active host subversion and inhibits host cell death categories, which is a hallmark of EHEC strains (Ho et al., 2013). IPEC strains also elicit aggressive adherence functions to enable pathogenicity (Kaur et al., 2010), but our methods did not enable clear emphasis of this category in pathogenic strains compared to nonpathogenic stains, likely due to the ubiquitous nature of adherence functions.

For Burkholderia, our analysis enables good separation, with the exception of B. pseudomallei K96243, a pathogen that clusters with non-pathogens (Figure 4B). Previous analysis of the genome of this strain noted high similarity to Ralstonia solanacearum, a plant pathogen (Holden et al., 2004), which is consistent with this strain clustering with B. glumae and B. phytofirmans (plant colonizers) in our analysis. *B. mallei* and *B. pseudomallei* are intracellular pathogens that use numerous virulence factors that enable host cell survival, such as invasion and immune evasion factors (Galyov et al., 2010; Memisevic et al., 2014), which is apparent in Figure 3B. These organisms also contain key factors such as BimA, hemagglutinin, PilA, which are involved in invasion, damage, and adherence, respectively (Sarovich et al., 2014) that enable emphasis of these categories in the plot. In contrast to *B. mallei* and *B. pseudomallei*, the only enriched functions for *B. cenocepacia* are antibiotic resistance and non-toxin damage functions, but this may be an indication of lack of coverage in our database (only 2 of the 141 hazardous Burkholderia functions are from *B. cenocepacia*). However, this finding is consistent with the fact that *B. cenocepacia* clinical strains isolated from cystic fibrosis patients can be resistant to antibiotics and contain several lipases and proteases to illicit tissue damage (Mahenthiralingam and Vandamme, 2005). Noticeably, *B. glumae* (third row from the bottom in Figure 3B demonstrates some pathogenic signatures, which is consistent with research demonstrating that this species can be a rice pathogen (Pedraza et al., 2018). This species was originally considered a nonpathogen based on the dataset published by Cosentino et al. (Cosentino et al., 2013), suggesting that our methods may enable identification of misannotated organisms.

Finally, some separation is also apparent for Pseudomonas species, but the patterns are not as consistent across strains as the other pathogens (Figures 3, 4F). Pseudomonas species pathogenic to humans (*P. aeruginosa* and *P. mendocina*) have a wide variety of virulence factors (Goldberg, 2010), but the patterns are different between the two species, and these two groups are completely separated in the dendrograms (Figure 4). For example, both *P. aeruginosa* and *P. medocina* have several proteins contributing to adherence and motility (Supplementary Table S1), but these types of functions can occur in nonpathogenic species as well. In contrast, invasion factors, host cell subversion factors, host cell apoptosis, and damage factors are relatively unique to *P. aeruginosa* strains (Figure 3 and Supplementary Table S1), which is consistent with experimental evidence (Shaver and Hauser, 2004; Dulon et al., 2005; Casilag et al., 2016; Basso et al., 2017; Reboud et al., 2017). Antibiotic-resistance functions are higher in *P. aeruginosa* pathogenic strains as well, which is consistent with the clinical prevalence of antibiotic resistant strains (Jacoby and Munoz-Price, 2005). For plant pathogens, our methods result in some separation of *P. syringae*—a plant pathogen—from nonpathogenic Pseudomonas species overall (Figure 4), and within the inhibits host cell death functional category (Figure 3). These observations may be driven by the fact that only 2 of the 175 Pseudomonas hazardous functions contained in our database are from *P. syringe.*

### Toward application of the methodology and resulting functional hazard database

**FIGURE 5:**
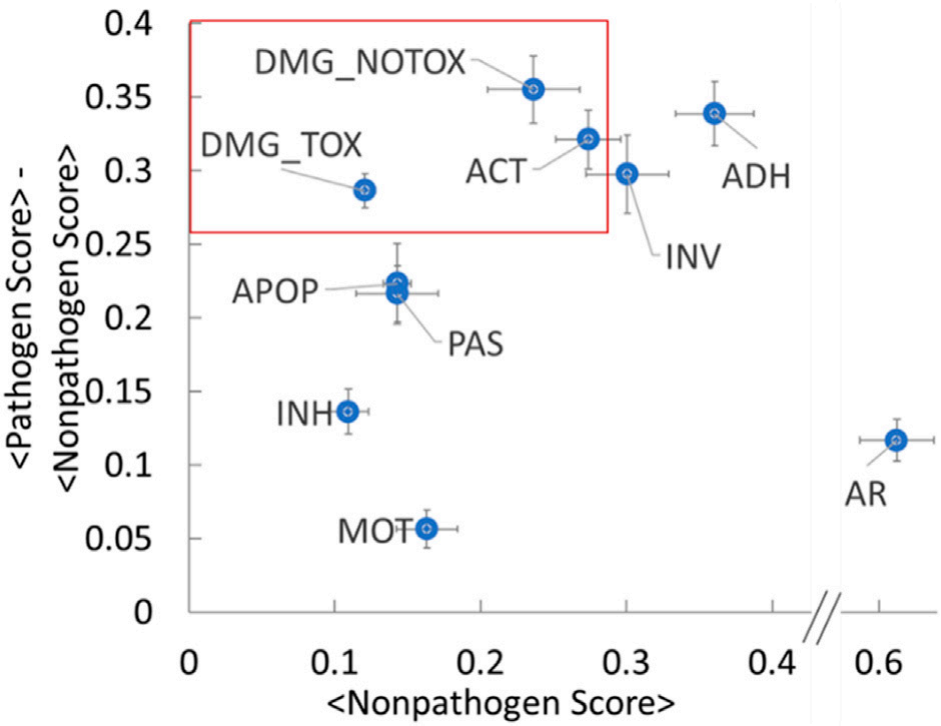
Pathogen and Nonpathogen Fingerprint Scores Reveal Stratification Among Functional Hazard Categories. The plot shows a difference in average scores between the pathogens and nonpathogens (*y*-axis) as a function of the average nonpathogen score (*x*-axis) for the functional categories from the heat plots in Figure 3. The error bars represent the standard error. The abbreviations are the same as Figure 3.

The fingerprint analysis presented in the previous section demonstrates that categorizing hazardous functions allows the importance of the gross functionalities (i.e., the functional metadata categories in Table 1) to differentiate nonpathogenic groups from pathogenic groups for both gram-negative and gram-positive bacteria. As further demonstration of our methodology and database with an eye toward the utility of our method for biosafety assessments, we sought to determine the relative hazard level of each functional category. Logic suggests that two parameters play a large role in such a relative ranking: 1) the magnitude of the category’s increase in relative abundance compared to nonpathogens and 2) the relative abundance of the category in nonpathogens. As a simple measure of these parameters, we leverage the data used to generate the heat plots to calculate an average score for each of the functional categories for the nonpathogen and pathogen groups. Figure 5 shows a plot of the difference in average scores between the pathogens and nonpathogens as a function of the average nonpathogen score. The points on the upper left quadrant of this graph thus represent highly hazardous categories that 1) have a relatively large difference between the pathogen and nonpathogen scores and 2) have a low background signature (i.e., low nonpathogen score). For example, these results suggest that the damage (with and without toxin activity) and active host subversion categories have relatively high pathogen-nonpathogen difference scores (e.g., >0.25) with low nonpathogen scores (e.g., <0.3) (red box in Figure 5). Such an analysis demonstrates a potential ranking system for “sequences of concern,” and may enable a foundation for a risk-based approach for biohazard assessments for designed organisms. As mentioned above, more hazardous functions that do direct damage to a cell or those involved in avoiding the host immune system rank more highly than less hazardous functions such as adherence and motility. Thus, the damage and active host subversion categories may present a higher hazard relative to other categories for biohazard analysis, for example. Generalizing this approach across all functional categories and all organism types may provide an objective foundation for biohazard analysis of novel organisms.

## Discussion

While the methodology and database presented here has two immediate uses—1) biosecurity screening assessments of synthetic genes and 2) partial biosafety assessments for bacterial genomes—future work should build upon this foundation to provide comprehensive biosecurity and biosafety assessments for the synthetic biology community. We envision a future in which any novel biodesign can be assessed through a function-based paradigm that requires only genomic sequences. This paradigm is in contrast to current biosafety assessments that rely on phenotypic information from well characterized organisms to classify organisms into Biosafety Levels, for example, which provides researchers with an understanding of the level of pathogenicity, transmissibility, and other characteristics of the organism (U.S. Department of Health and Human Services, 2014). However, as the genomes of new biodesigns begin to deviate further and further from these well characterized organisms, biosafety levels become less and less clear, thus necessitating in silico genome characterization methods. Where traditional biosafety assessments are limited to known pathogens with no or minimal bioengineered parts, with future development, our framework may enable assessment of seemingly limitless potential for biodesigned organisms. In this discussion, we elaborate on the issues with the current paradigm, how our approach begins to shift the paradigm, and the future work needed to provide a complete paradigm shift.

Progress in bioengineering, synthetic biology, and computational science is enabling artificial creation (*de novo* genetic synthesis) of whole organisms, including viruses (Blight et al., 2000; Cello et al., 2002; Smith et al., 2003; Oldfield et al., 2017; Noyce et al., 2018) and bacteria (Gibson et al., 2010; Hutchison et al., 2016), as well as recombinant production, viral reverse genetics, rational design, design from standardized DNA components (e.g., Biobricks), and/or modular protein assembly (e.g., SpyTag or SpyCatcher (Khairil Anuar et al., 2019)). Such technologies have led to exponential growth of publications based on synthetic biology since 2000, and larger throughput per synthetic biology lab (Raimbault et al., 2016). Further yet, DNA synthesis is becoming more distributed, for instance, with the availability of DNA printers such as the BioXp system from Codex DNA. As breakthroughs are made to realize the promise of synthetic biology, the creation of novel sequences may expand even more, and such growth is difficult to monitor. Although the numbers of new natural strains being discovered is accelerating fairly linearly (Suzek et al., 2015; RefSeq, 2019), the production of bioengineered strains may be growing exponentially, as many of these sequences are not publicly available. This rapid progress in bioengineering has created a gap in current biosafety practices that requires a framework to understand the potential hazards posed by functional building blocks. We have provided empirical data that demonstrates a function-centric paradigm for identifying and classifying hazardous biological parts. The functional classification of sequences is based on coarse hazardous functions encoded by organisms, such as functions contributing to pathogenicity, toxin and drug production, and immune regulation.

The methodology demonstrated here can immediately be used for partial biosafety assessments for bacterial genomes for classification of pathogens and non-pathogens using functional hazard fingerprints. Future iterations of the method should involve testing both previously characterized organisms and novel organisms (i.e., those not contained in the database and/or novel biodesigns with known phenotypes) in order to characterize a variety of biosafety-related characteristics (not just pathogenic/nonpathogenic) from various domains of life beyond bacteria (e.g., viruses and fungi). As we demonstrated in Figure 4, hierarchical clustering achieves a high level of separation between pathogen and nonpathogen organism group members using a simple alignment with default parameters against our curated database. This approach is in contrast with more complicated, manual annotation and phylogenetic analysis that require time-consuming, expert interpretation. Even outlier pathogens that cluster with nonpathogens like *S. suis ST3* have characteristics that explain why they do not cluster with other pathogens; for example, as noted *S. suis ST3* clusters with nonpathogenic organisms but is missing a pathogenicity island, which likely contains several hazardous functions. Similarly, outlier nonpathogens that cluster with pathogens such as *B. cereus Q1* can be explained as well. The genome for this organism contains genes encoding for enterotoxins (NCBI accessions ACM12308, ACM12309, and ACM12310) involved in damage and adherence, lipid transferases involved in passive and active immune subversion (accessions ACM11963 and ACM12924) and antibiotic resistance (accessions ACM12845 ACM12455). Thus, if used for assessments of pathogenicity, false negatives (due to lack of hazardous functions and/or presence of previously uncharacterized functions) and false positives (due to the presence of hazardous pseudogenes and/or non-hazardous sequences with high homology to hazardous functions) could occur depending on the thresholds used for classification. However, the success of this approach demonstrates the native utility of the hazardous function database and that further refinements in fingerprinting approach are both attainable and could be an effective diagnostic approach to classifying unknown organisms.

As documented in Table 3, some pathogens have higher coverage in our database than others, and thus comparison across pathogenic groups should be interepreted appropriately. Differences between a pathogen and nonpathogen in one organism being less pronounced relative to another organism group could be due to large functional differences, but it could also be due to lack of database coverage. For example, the fact that *M. tuberculosis* pathogens have higher numbers of hazardous functions compared to *N. gonorrhoeae* does not mean necessarily that *N. gonorrhoeae* is relatively more pathogenic compared to its nonpathogenic conterparts than *M. tuberculosis*; this result may be driven by the larger coverage of Mycobacterium sequences within our database. Determination if our approach can be used to elucidate levels of pathogenicity based on a collection of hazardous functions warrants further exploration. Such an application may have utility beyond biosafety assessments, such as emerging and recurrent disease identification. As recently stated by others, new approaches are needed to address emerging diseases (Reperant and Osterhaus, 2017), particularly as surveillance and diagnostics improve across the globe. We propose that a function-based paradigm provides a foundation to meet this need, and such approaches have already shown success. In this study, we leveraged data from Cosentino et al., who developed methods to classify bacterial pathogens from nonpathogenic bacteria based on protein families (Cosentino et al., 2013), which have a direct link to function (Pearson, 2013). Beyond bacteria, others have shown that sequence differences leading to functional differences are critical determinants of pathogenicity for viruses and fungi such as influenza virus (Ebrahimi et al., 2014; Straus and Whittaker, 2017), African Swine Fever Virus (Chapman et al., 2008), Zika virus (Shah et al., 2018), Colletotrichum spp. (Vieira et al., 2019), and Geosmithia spp. (Schuelke et al., 2017). Thus, development and generalization of models may aid in the shift from organism to function-based classifications for all types of infectious disease. For example, a logical extension of the study presented here would be to determine if similar results can be obtained if we leveraged our entire database (not just specific subsets of hazardous functions from selected bacteria), such that a prior knowledge of the organism in question is not needed.

In addition to the immediate use of our methods for predicting pathogenicity of bacteria, the method and database also has immediate use for screening individual gene sequences. The example application of our methodology and database to stratify sequences are promising, but the results suggest more granular functional categories may be needed to enable use for more pointed biosafety assessments. Granular metadata for protein sequences are available from several databases that are cross-referenced within the UniProt Knowledge Database (UniProt, 2019b), such as Gene Ontology (GO) terms (Ashburner et al., 2000; The, 2019), Interpro terms (Mitchell et al., 2019) and sequence features (e.g., motifs, regions, mutation impact, etc.). GO terms provide a graphical representation of molecular functions, biological processes, and cellular components of gene products and their relations among each other (Ashburner et al., 2000; The, 2019). We leveraged the “toxin activity” GO term within our framework, but further use of GO terms may enable better stratification of hazardous sequences.

Our results may also improve if host information is considered. Recent efforts, such as ViralZone (ViralZone, 2019) and the proposed PathGO (IARPA. Broad Agency Announcement, 2016) are providing better GO terms for host-pathogen interactions that may prove valuable for function-based hazard classification. Casadevall proposed a damage response framework (Casadevall and Pirofski, 2003) that is founded on the simple principle that microbial pathogenesis is “the outcome of an interaction between a host and a microorganism” measured by damage to the host. Current knowledge suggests pathogens interact with the host in a variety of ways, including mimicking host activities, leading to a lack of host cellular control (Knodler et al., 2001; Stebbins and Galan, 2001; Smatti et al., 2019), but documentation of these data in a machine-readable format is sparse. Two potentially useful sources of information that are cross-reference in UniProt are IntACT (Hermjakob et al., 2004), which provides protein-protein interaction data, and Reactome (Reactome, 2019), which provides functional metadata associated with biological pathways. An initial analysis of our hazardous functions suggests that <2% of protein accessions in our database have at least one interactor in IntACT database, and 58% of the interacting proteins are human proteins. These human proteins represent 3% of the total reactome metadata. In addition to IntACT, specific (host-pathogen) protein-protein interaction information is available from Biogrid (Oughtred et al., 2019), String (von Mering et al., 2005) and other databases, but information is sparse. However, as high-throughput experimentation becomes more commonplace, information contained in these databases can be leveraged for hazard analyses. Specifically, further expansion of these databases for hazardous sequences may be needed for impactful analysis and utility into a function-based biosafety assessment.

In addition to hazards that may impact hosts such as humans, livestock, and crops, other living hosts and non-living “hosts” of economic importance should be considered as well for other pointed biosafety assessments. For example, when considering safety assessments for novel bio-based fertilizers and/or biopesticides, hazards with economic impact potential beyond those that effect crops and livestock may need to be considered. For example, of the world’s ∼250,000 flower and seed-producing plant species, between 78% and 94% require pollinators for fertilization (“FAOSTAT” Food and Agricultural Organization www.fao.org), with bees accounting for pollination of approximately 30% of the world’s food supply (Klein et al., 2007). Bee colonies can collapse from fungal, bacterial and viral outbreaks, such as those caused by the picornavirus-like deformed wing virus (DWV) and the ectoparasitic mite Varroa destructor (Tehel et al., 2019). Similarly, functions that could negatively impact non-eukaryotic or non-living “hosts” of economic importance should also be considered for tailored safety assessments. For example, under the current paradigm of biosecurity, biodesigns have been created that could potentially impact biomanufacturing supply chains (Abdulamir et al., 2014), control of pharmaceuticals (Galanie et al., 2015; Nakagawa et al., 2016), and crude oil supplies (Xu et al., 2018). Thus, as bioengineering rapidly progresses, safety practices need to keep pace to not only protect humans, livestock, and crops, but also protect infrastructure of critical economic impact.

Expansion of sequences and metadata may thus improve upon our foundation for biosafety practices of the bioengineering-centric future. Our methods and database reported here provide an understanding of the hazard posed by “parts” of the organism, such that a foundation can be set to understand the hazard of the “whole.” For example, *P. aeruginosa* has numerous hazardous functional parts including those contributing to adherence (type 4 pili and flagella for interacting host cells), invasion (T3SS), host cell subversion (biofilm formation, stimulation of proinflammatory response, and disabling of protease activity receptor-2), host cell apoptosis (exotoxin A stimulation of programmed cell death), damage (and cytotoxic effector proteins) and antibiotic resistance (beta-lactamases) (Shaver and Hauser, 2004; Dulon et al., 2005; Jacoby and Munoz-Price, 2005; Casilag et al., 2016; Basso et al., 2017; Reboud et al., 2017; Shen et al., 2017). While many of the hazardous functions of *P. aeruginosa* are known, a biodesign created with similar hazardous functions may not be identified under the current organism-centric paradigm. We must now build upon our methods developed using the engineering-like principle of pathogens being an organized assembly of functional hazards. Using this paradigm, we can then classify groups of sequences that compose a novel pathogen, thus enabling generalized function-based biosafety assessments for novel organism-level biodesigns for all types of applications.

## Methods

### Hazardous function database

Hazardous functions were identified from publicly available literature and databases (e.g., Supplementary Table S2) as those that have a function that impacts human and non-human hosts of high economic value as described in the Results section. We defined a hazardous function as a set of one or more protein sequences and associated manually curated metadata (Table 1). Each hazardous function can contain one or more functional categories. A hazardous function is only included in the database if its sequence encodes for a verified function based on experimental data from the literature or (in cases such as some select agent viruses where experimental data do not exist) based on homology to a sequence with verified function. Protein sequences were retrieved from UniProt when available or manually entered based on literature documentation. Functional metadata categories were developed based on panel discussions of high-level hazardous functions used by pathogens and organisms producing toxins, drugs, and bioregulators. For hazardous functions in the “damage,” category, the toxin activity gene ontology term (GO:0090729) was used to distinguish toxins from non-toxins. Further, for sequences involved in the biosynthesis of small molecule toxins or drugs, hazardous functions were annotated with the step removed from the final product (e.g., last step, second-to-last step) based on pathway information as described in the literature and/or on Metacyc (Caspi et al., 2018).

### Identification of hazardous coding sequences from bacteria

To validate the above methods and resultant database, we compared pathogenic and non-pathogenic strains against our functional hazard database. For this exercise, we compiled coding sequences (CDSs) from human and animal pathogenic and nonpathogenic strains based on the references outlined in Table 3. For each identified reference, pathogenic and nonpathogenic strains were reviewed; if a nonpathogenic strain was revealed as a pathogenic strain to a host of interest (or vice versa) based on other literature sources (e.g., a source published after the primary reference), it was removed from the analysis. Further, if an organism has known plasmids with sequences not deposited in NCBI, it was removed from the analysis. Pathogenic species or strains from each organism group were further stratified into subgroups based on species groups or disease-causing metadata (Table 3, column 4) for comparative purposes. CDSs, including those from chromosomal accessions and associated plasmid accessions were downloaded using NCBI’s Batch Entrez online tool (NCBI, 2019). Plasmids were included since genetic determinants of bacterial virulence are often carried on mobile elements such as transposons and plasmids (Zaluga et al., 2014). Each strain’s CDSs were defined by those contained within all chromosomes and plasmids associated with that strain. For each organism group, CDSs were aligned against a database of hazardous functions from its same genus using the Local Aligner for Massive Biological DatA (Lambda) (Hauswedell et al., 2014) version 2–1.9.5 using default settings. The alignment score, A , was defined as

A=Percent Identity×Percent Hazardous Sequence Coverage (1)

As discussed, we define the minimal alignment score for a CDS to be a hazardous function as 40% based on the thresholds used to define UniRef50 clusters. We then determined the fraction of hazardous CDSs (total number of CDSs in each strain normalized by the strain’s total number of CDSs) and averaged the results of each strain within each pathogen and nonpathogen group.

### Hazardous function fingerprinting

To determine a hazard function fingerprint for each strain, the alignment scores, A , for each CDS (to the genus-specific hazardous function database) were summed for each functional category then normalized to the maximal value across all pathogen and nonpathogen groups within that functional category. If a strain did not have a CDS with an alignment to the hazardous function, A was set to zero. Since each hazardous function can contain one or more functional categories, we defined the fingerprints as follows. For each CDS set of alignment results (i.e., one CDS to one or more hazardous functions), the maximal A for each functional category (Table 1) was tabulated. For example, suppose ${CDS}_{i}$ aligns to ${Hazardous\;Function\;Sequence}_{1}$ and ${Hazardous\;Function\;Sequence}_{2}$ with an A of 1.0 and 0.8, respectively. If ${Hazardous\;Function\;Sequence}_{1}$ has adherence metadata and ${Hazardous\;Function\;Sequence}_{2}$ has both adherence and invasion metadata, the fingerprint score contribution for ${CDS}_{i}$ would be 1.0 for adherence and 0.8 for invasion. Maximum A scores for each functional category for each strain were then summed across each strain’s CDSs. The final fingerprint score for each strain was defined as the cumulative A within each category normalized by the strain’s total number of CDSs then normalized by the maximal value across all pathogen and nonpathogen strains within that functional category.

Hierarchical clustering analysis was performed in R using the function hclust, with UPGMA as the method for agglomerative clustering. Dendrograms were plotted using the R libraries ggdendro and ggplot2.

## Data Availability

The original contributions presented in the study are included in the article/Supplementary Material, further inquiries can be directed to the corresponding author.

## Footnotes

1 For this manuscript, the term biosafety refers to practices associated with protecting researchers from biological hazards associated with an organism based on its characteristics (e.g., practices associated with Biosafety Level 3 organisms). The term biosecurity refers the security of biological materials, including ordering of synthetic nucleotides. Thus, understanding the hazards associated with single synthetically made sequences can aid in biosecurity assessments (i.e., fulfilling synthetic nucleotide orders), whereas understanding the pathogenicity of an organism being manipulated in a laboratory can aid in biosafety assessments.

## Authors note

The authors have carefully reviewed and discussed the concepts in this manuscript for dual use concerns both internally as well as with members of the US Government, the International Gene Synthesis Consortium (IGSC), and Engineering Research Council (EBRC). While we understand the risks, the prevailing opinion is that the methodologies presented here in themselves do not provide a roadmap for creation of harmful organisms, nor do they enable circumvention of screening. In fact, this manuscript provides the scientific community a potential framework for screening, which should help improve biosecurity through improved screening practices. Further, the authors purposefully did not publicize our database to further alleviate such concerns.

## Supplementary material

Hazardous Functions Partially Separate *E. coli* Pathogen Groups Shown are the dendrograms for *E. coli* grouped by type of *E. coli*. Pathogenic species colored as follows: EHEC (red), ExPec/UPEC (purple), EAEC/ETEC/AIEC/EPEC (orange). Non-pathogenic species are colored as follows: commensal (green and teal) and yellow (lab strains).
