# Supplementary material for: Function-based classification of hazardous biological sequences: Demonstration of a new paradigm for biohazard assessments
Source: Front Bioeng Biotechnol. 2022 Oct 7;10:979497. doi: 10.3389/fbioe.2022.979497 (PMC9585941; doi:10.3389/fbioe.2022.979497)
Supplement: Supplementary file 1 [file DataSheet1.docx]

**Supplemental**

Table S 1 Specific Hazardous Functions Enriched in Pathogenic Groups

| Pathogenic Group | # Highly Confident Hazardous Functions Unique to Pathogenic strains* | Example hazardous functions (Exemplar Uniprot Accession) | Metadata* |
| --- | --- | --- | --- |
| *N. gonorrhoeae* | 3 | IgA-protease (P09790), Outermembrane protein C (Q51227), Factor H binding protein (Q6VS32), Adhesion proteins (Q9JXL6) | Act, Inv, Pas, Adh |
| *N. meningitidis* | 12 | Capsule proteins (P0A0Z7), Outermembrane protein PorA (P0DH58), Factor H binding protein (Q6VS32), Adhesion proteins (Q9JXL6 | Act, Adh, Pas, Inv |
| *E. coli* IPEC: EAEC/ETEC/AIEC/EPEC | 0 | -- | -- |
| *E. coli* IPEC: EHEC | 50 | Shiga-like toxin (P09386), Effector proteins (A0A376U116), T3SS proteins (A0A2D0NWR6), | Dmg, Act, Adh, Inh, Inv |
| *E. coli* ExPEC | 9 | Pilus proteins (P62532), Glycoporin (A0A0F6CBL1), fimbria adhesin (P13429) | Act, Adh, Inv |
| *B. mallei* | 52 | Capsule enzymes (H7C745), TSSS proteins (Q63K26), Effector proteins (Q63K42), Beta-lactamase (A0A0E1UDG8), BimA (B4Y9V9) | Dmg, Act, Adh, Pas, Apop, Inv, Ar, Mot |
| *B. pseudomallei* | 54 | Capsule enzymes (H7C745), TSSS proteins (Q63K26), Effector proteins (Q63K42) | Dmg, Act, Adh, Pas, Apop, Inv |
| *B. cenocepacia* | 4 | Metalloprotease ZmpA (Q2VL23), Antibiotic resistance kinase (A0A0H2Y028), Capsule proteins (Q63R84) | Dmg, Act, Ar |
| *P. aeruginosa* and *P. mendocina* | 23 | Multidrug resistance protein (P52003), Hemolytic Phospolipase C (P06200), Adhesin proteins (Q9HVM8), effector proteins (A0A0H2Z790), Protease IV (Q9HWK6) | Dmg, Act, Adh, Inh, Mot, Pas, Inv, Apop, Ar |
| *P. syringae* | 1 | Sigma factor (G3XCZ2) | Act |
| *S. pneumoniae* | 14 | Pneumolysin (Q04IN8), Adhesin proteins (Q8CYC9), Immunoglobulin A1 protease (B2IPX1), cell wall enzymes (Q8DP63) | Dmg, Act, Adh, Pas, Ar |
| *S. pyogenes* | 25 | Streptolysin S (Q48UF2), exotoxin B (P0C0J0), IgG-degrading enzyme (Q9F1R7) | Dmg, Act, Adh, Mot, Inv |
| *S. suis* | 7 | IgG-degrading enzyme (A5JSJ3), Suilysin (T1R445) | Dmg, Act, Inv |
| *B. cereus* and others (See Table 3) | 14 | Enterotoxins (Q09KI4), Phospholipase (O52864), Immune inhibitor (P23382) | Dmg, Act, Adh, Pas, Inv, Ar |
| *B. anthracis* | 25 | Protective antigen (P13423), Edema factor (P40136), Lethal factor (P15917), Cereolysin (Q531A4) | Dmg, Act, Adh, Pas, Inv, Ar |
| *C. botulinum* and *C. tetani* | 2 | Clostridiolysin (A5HZ31), Botulinum toxins (P0DPI0) | Dmg |
| *C. difficile* | 3 | Hemorrhagic toxin (P16154), Virginiamycin A acetyltransferase (U5MQN2) | Dmg, Adh, Inv, Ar |
| *C. perfringens* | 4 | Collagenase (P43153), Alpha toxin (Alpha toxin) | Dmg, Act, Adh, Inv, Ar |
| *M. tuberculosis* and others (See Table 3) | 70 | Acid phosphatase (O53361), PE proteins (A0A045IS84), Adenylate cyclase toxin (O53213) | Dmg, Act, Inh, Inv, Ar |
| *M. leprae* and others (See Table 3) | 7 | Transmembrane invasion protein (A0A045JWB8), Alkyl hydroperoxide reductase (A0A0H3M8M8) | Inv |

*Act=active host subversion, Inv-invasion, Adh=adherence, Pas=passive host subversion, Dmg=damage, Apop=apoptosis, Mot=motility, Ar-antibiotic resistance, Inh-inhibits host cell death

Table S 2**.** Notable Databases of Virulence Factors, Toxins, and other Hazardous Functions

| Database type | Database | Example Hazards Contained in Database | Functional Metadata |
| --- | --- | --- | --- |
| Microorganisms | Virulence Factor Database (VFDB) [32] | Bacterial virulence factors such as those involved in adherence, invasion, or toxin activity of the bacteria | Functional classification tags (e.g., adherence, toxin, etc.) |
|  | Victor’s Virulence Factors [31] | Bacterial, viral, parasite and fungal virulence factors | Pathogen type (e.g., zoonotic), curator tags (e.g., “immune evasion”) and referenced assertions |
|  | Patric [105] | Bacterial virulence factors similar to the above two databases and annotated genomes | Classification tags (e.g., “defense against immune response”) and referenced assertions |
|  | Database of Fungal Virulence Factors (DFVF) [189] | Fungal virulence factors | Host information, disease tags, and assertions |
|  | ProTraits [190] | Microbial pathogenic phenotypes | Predicted microbial habitats, phenotypes, keywords |
|  | MvirDB (LLNL Database) [191] | Microbial toxins, virulence factors and antibiotic resistance genes | Unknown; not publicly available |
|  | Pathogen-host Interaction database (Phi-base) [192] | Fungal, Oomycete, and bacterial pathogens, which infect animal, plant, fungal and insect hosts | Disease, host, and mutant phenotype information |
|  | Pathogenicity Island Database [193] | Bacterial virulence and antibiotic resistance factors on pathogenicity islands | Functional tags (e.g., enterotoxin) and referenced assertions |
|  | Islander [194] | Bacterial virulence factors on pathogenicity islands | Island identifiers |
|  | ICEberg [195] | Bacterial transposable elements | Transferable hosts, functional assertions |
|  | Pseudomonas Genome Database [196] | Pseudomonas virulence factors | Functional category, host organism, and evidence codes |
|  | Comprehensive Antibiotic Resistance Database (CARD) [197] | Antibiotic resistance genes and phenotypes | Ontology terms and references |
| Viruses | Virus-Host Database [198] | Pathogenic virus proteins | Virus hosts and evidence |
|  | Viral Zone [199] | Pathogenic virus proteins | Geographical information, associated diseases, transmission, host information |
|  | Influenza Research Database [200] | Influenza proteins | Sequence features (e.g., nuclear localization motif), epitopes, motifs, keywords (e.g., host-virus interaction), variant information, references |
| Toxins | Toxin and Toxin target database (T3DB) [201] | Toxins of all types (proteins, small molecules, inorganic, etc.) | Toxin category (e.g., food toxin), mechanism assertions, targets |
|  | Animal toxins in UniProt [202] | Animal toxins secreted in animal venom | Gene ontology terms, Interprot terms, and other metadata provided by Uniprot |
|  | Kalium [203] | Potassium channel toxins from scorpion venoms | Toxicity dose, host channel subtype |
|  | Conoserver | Conopeptides (conotoxins and closely related toxic peptides) | Class, pharmacological family, organism information (clade, geographical information), cysteine framework |
|  | ISOB [204] | Toxins in snake venoms from Bangladesh | Geographical distribution of snakes, toxin class (e.g., neurotoxin), references |
|  | ArachnoServer [205] | Protein toxins from spider venoms | Toxin group, host molecular target, binding properties |
| Prions | PrionHome [206] | Prions and related proteins | Unknown; link no longer available |
|  | PrionScan [207] | Prion domain prediction | Prion domain prediction score |
| Small molecules pathways | Biosynthetic pathways from Metacyc [182] | Biosynthetic pathways for toxins and other small molecule hazards | Pathway identifiers, pathway types, and referenced assertions |
|  | Small Molecule Pathway Database [42] | Pathways of small molecule toxins | Host small molecule targets, descriptions, and references |
|  | Reactome [208] | Pathways, reactions, and binding events associated with toxin uptake, virus entry, etc. | Molecular events (e.g., uptake of bacterial toxins) References, |
| Bioactive Peptides and Drugs | DrugBank [209] | Drug targets, enzymes, and transporter for biosynthetic-derived approved and illicit drugs (e.g., nicotine) | Metabolite-protein interactions, drug description, drug group (e.g., illicit, approved) |
|  | Milk Bioactive Peptide database [210] | Peptides that bind opioid receptors | Peptide functions (opioid, ACE-inhibitory, immunomodulatory) and references |
|  | BioPepDB [211] | Immunomodulatory and opioid-like peptides | Functional category (e.g., opioid), source organism categories, references |
|  | Endogenous Regulatory OligoPeptides [212] | Peptide and immunomodulatory peptides | Functional category (e.g., hormone, toxin), comments, and references |
|  | Hemolytik [213] | Peptides with hemolytic activity | Functional category (e.g., toxic, anti-fungal), source, activity |
|  | PEPlife [214] | Hormones, blood coagulation inhibitors | Biological activity, half life |
|  | StaPep [215] | Toxin peptides, neuropeptides | Peptide functional class |
|  | THPdb [216] | Immunosuppressing peptides, hormones | FDA*-approved use (e.g., cosmetics), disease type |

*FDA, Food and Drug Administration

Table S 3. Example Protein/ Peptide Bioregulator Hazardous functions

| **Bioregulator** | **Produced by** | **Host System Impacted** | **Hazardous Function** |
| --- | --- | --- | --- |
| Dermorphin/ Deltorphin | Amphibians | Nervous System | Opiate-like activity (>30X more potent than small molecules opioids) [217] |
| Anaphylatoxins | Mammals | Immune and other system | Facilitates inflammatory processes, vascular permeability, and contraction of smooth muscle.  [217] |
| Protachykinin (substance P) | Mammals | Nervous, immune, and cardiovascular systems | Disturbs short-term memory, creates anxiety/ depression [218]; helps create inflammatory response [217]; causes extreme vasodilation [219] |
| Bradykinin | Mammals | Cardiovascular and muscular system | Vasodilation, contraction of smooth muscle  [217] |
| Interleukins | Mammals | Immune system | Regulates immune response, can cause inflammation |
| Galanin | Mammals | Nervous system | Impedes new memory formation, the formation of new memories, regulates nociception [217] |

Table S 4. Example Protein Toxins Hazardous functions

| **Cellular Process** | **Target** | **Native Production host** | **Activity** | **Example Hazardous functions (organism)** |
| --- | --- | --- | --- | --- |
| Protein synthesis | Ribosomes | Plants, Bacteria | Cell surface binding, N-glycosidase activity [220; 221; 222] | Ricin (*Ricinus communis*), abrin (*Abrus precatorius*)*;* Shiga toxin (Shigella dysenteriaei), verotoxins/Shiga-like toxins (E. coli) |
| Protein synthesis | Elongation factor (EF-2) | Bacteria | Receptor binding, ADP-ribosyltransferase* [222; 223] | Diphtheria toxin (*Corynebacterium diphtheriae*), exotoxin A (*Pseudomonas aeruginosa*), Cholix toxin (*Vibrio cholerae*) |
| Actin polymerization | Actin or Rho family of GTPases | Bacteria | Receptor binding, ADP-ribosyltransferase or Glucosyltransferase* [224] | Binary toxin, Toxin A and B (*Clostridium difficile*), iota toxin (*Clostridium perfringens*) |
| Cellular structure | Claudin | Bacteria | Claudin binding to stimulate host cell pore formation [225] | Enterotoxin (*Clostridium perfringens*) |
|  | Phospholipids, Calcium ions, or coagulation proteins | Bacteria, reptiles | Phospholipase (PL)-based or protease-based hemolysis and/or platelet-aggregation [226; 227; 228; 229] | PL-C (*Clostridium sp.*, *Bacillus sp., B. pseudomallei*), PL-A2, PL-D, or serine proteases (snakes, spiders, scorpions) |
| Cell cycle | Double stranded DNA | Bacteria | Receptor binding, Dnase ** [230] | Cytolethal distending toxin (*Campylobacter sp,* *E. coli,* etc.) |
| Cell signaling | Heterotrimeric protein G (cyclic AMP production) | Bacteria | Receptor binding, ADP-ribosyltransferase* [222] | Cholera toxin (*Vibrio cholera*), Pertussis toxin (*Bordetella pertussis*); heat-labile enterotoxin A (*E. coli*) |
|  | Guanylate cyclase C (cyclic GMP production) | Bacteria | Guanylate cyclase C binding [231; 232] | Heat-stable enterotoxin (*E. coli*) Enterotoxin A (*Yersinia enterocoliticai*) |
|  | Adenosine triphosphate (ATP) | Bacteria | Adenylate cyclase [115; 126; 222] | Adenylate cyclase (*B. anthracis, M. tuberculosis,* etc.) |
| Cell and Immune signaling | ATP + mitogen-activated protein kinase (MAPKK) | Bacteria | Receptor binding + Adenylate cyclase + Metalloprotease, impacts regulatory molecules [115] | Anthrax toxin: protective antigen + edema factor + lethal factor (*B. anthracis*) |
| Immune signaling | Major histocompatibility complex (MHC) II | Bacteria | MHC class II binding, inflammatory response [233] | Superantigens, toxic shock syndrome toxin (*Staphylococcus aureus*) |
| Neuromuscular signaling | SNARE proteins | Bacteria | SNARE protein cleavage | Botulinum neurotoxins (*C. botulinum*), tetanus neurotoxin (*C. tetani*) |
| Pore formation | Erythrocytes cell membranes and receptors | Bacteria | Receptor binding, membrane binding [234; 235] | Cytolysin A (*E. coli*), alpha-hemolysin (*Staphylococcus aureus*) |
| Neuronal and other signaling | Various ion channel and G-coupled receptors and transporters | Various animals | Receptor agonist/ antagonists (e.g., acetyl-choline receptors) [228] | Conotoxins (*Conus sp.*), beta toxins, omega-agatoxins, and other animal toxins (scorpions, spiders, snakes, etc.) |

* AB toxin in which A is catalytic subunit, B is binding subunit.

** Heterotrimeric toxin “AB toxin” in which CdtB is the catalytic subunit, whereas CdtA and CdtC make up the binding subunit.


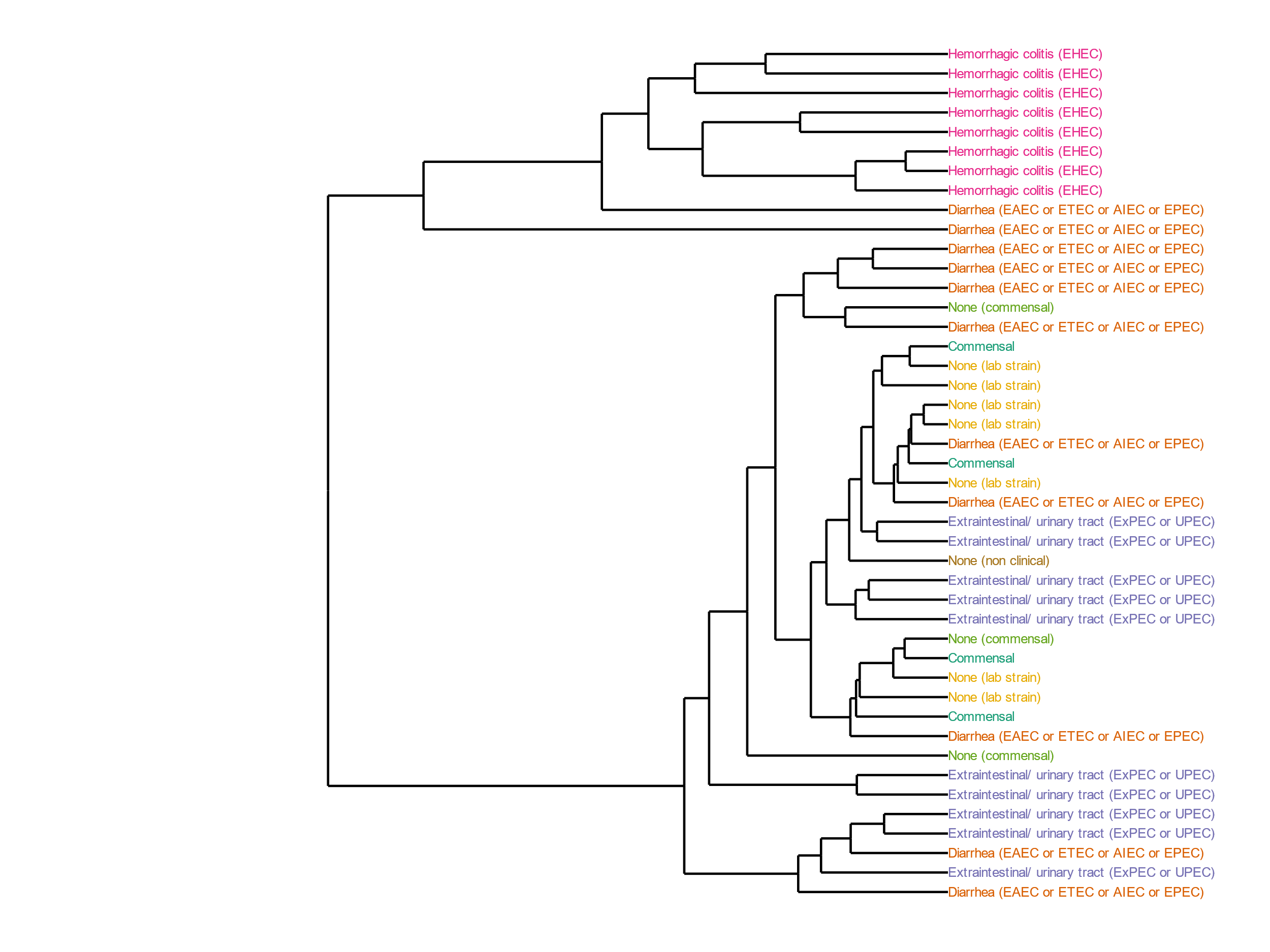


***Supplementary Figure 1. Hazardous Functions Partially Separate E. coli Pathogen Groups***

*Shown are the dendrograms for E. coli grouped by type of E.coli. Pathogenic species colored as follows: EHEC (red), ExPec/UPEC (purple), EAEC/ETEC/AIEC/EPEC (orange). Non-pathogenic species are colored as follows: commensal (green and teal) and yellow (lab strains).*
